# Mean platelet volume as a predictive marker for venous thromboembolism in patients treated for Hodgkin lymphoma

**DOI:** 10.18632/oncotarget.25002

**Published:** 2018-04-20

**Authors:** Joanna Rupa-Matysek, Lidia Gil, Marta Barańska, Dominik Dytfeld, Mieczysław Komarnicki

**Affiliations:** ^1^ Department of Haematology and Bone Marrow Transplantation, Poznan University of Medical Sciences, Poznań, Poland

**Keywords:** Hodgkin lymphoma, mean platelet volume, venous thromboembolism, Khorana Risk Score, ThroLy Score

## Abstract

Mean platelet volume (MPV) is reported to be associated with the risk of venous thromboembolism (VTE) and mortality in patients with cancer.

We sought to determine the association of MPV with symptomatic VTE occurrence in patients treated for newly diagnosed Hodgkin lymphoma (HL) and their outcomes. We retrospectively studied 167 consecutive adult patients treated with HL. During first-line treatment 12 (7.2%) patients developed VTE and 14 (8%) died within the observation period. The pre-chemotherapy values of MPV were significantly lower in VTE patients than those without (p=0.0343). Patients with MPV≤25^th^ percentile (6.8 fl) had an increased risk of VTE occurrence (p=0.0244). In multivariate analysis, MPV≤25^th^ percentile (OR 2.21; 95%CI 1.07-4.57, p=0.033), advanced stage (OR 2.08; 95%CI 1.06-4.07, p=0.033) and bulky disease (OR 2.23; 95%CI 1.16-4.31, p=0.016) were significant factors for developing VTE. Only the impact of MPV≤25^th^ percentile on VTE-free survival rates was found. VTE occurred in 43% (n=3) of the high-risk patients of the Thrombosis Lymphoma (ThroLy) score and in 17% (n=2) of the high-risk of the Khorana Risk Score (KRS). Neither the KRS nor the ThroLy score could identify patients at a high risk of VTE with a high degree of accuracy. We expanded the ThroLy score with the addition of the MPV≤25^th^ percentile to more accurately identify HL patients with a higher risk of VTE.

Our study indicates that the pre-chemotherapy MPV value, while of no use as an overall prognosis predictor, may still represent a useful prognostic marker for a significant VTE risk especially when incorporated into VTE-risk assessment models.

## INTRODUCTION

Lymphoma patients are considered to be at a high risk of developing venous thromboembolism (VTE) [1]. It is known that tumour-associated VTE aggravates the clinical course of the disease, worsens the survival prognosis and contributes to death in cancer patients [2]. Recently, it has have reported that various biomarkers are predictive of VTE and mortality in cancer patients [3]. Some of these biomarkers were used in the development of predictive models for chemotherapy-associated thrombosis including the most common, the Khorana Risk Score (KRS), which unfortunately provides a low positive predictive value [4]. Moreover, some of these biomarkers are only used as research tools and there is a need for cheap and readily available parameters to predict VTE. Among other factors, platelet activation plays a role in cancer-associated thrombosis, as well as being a prognostic factor [5–8]. Recent studies have revealed that mean platelet volume (MPV), which is considered to be a marker of platelet activation, is associated with the risk of VTE in patients with cancer [9, 10]. Therefore, identification and incorporation of new variables in prognostic models associated with the risk of thrombosis may be valuable.

The aim of the present study was to determine the association of MPV with symptomatic VTE occurrence in patients treated for newly diagnosed Hodgkin lymphoma (HL). Moreover, we evaluated the impact of MPV on the outcomes of patients with HL.

## RESULTS

### Patient characteristics

One hundred and sixty seven adult patients with HD who underwent first line treatment were included in the study. All patients were white with a median age of 37 years (range 18-79 years), of whom 54% were females. The median observation time was 44 months (range 5-87).

The majority of patients were presented as advanced lymphoma (stage III and IV; n=88, 53%). On the International Prognostic Score (IPS), 63 cases had a score of 3-7 (high risk, 38%). Mediastinal involvement was observed in 26 cases (15.6%). None of the patients had obesity (BMI > 30 kg/m^2^) nor reduced mobility (ECOG 2-4). Only 4 patients had neutrophils below 1×10^9^/L. Two patients had previous VTE/acute myocardial infarction/stroke. Patient characteristics are provided in Table 1.

**Table 1:** Comparison of patients’ characteristics with or without VTE

Characteristic	Overall population n=167	Patients with VTE during follow-up^1^ n=12 (7.2%)	Patients without VTE during follow-up^1^ n=155 (92.8%)	p value
Median age, (range) years	36.7 (18-79)	35.0 (24-39)	37.7 (18-79)	0.2305
Gender, male n (%)	77 (46%)	6 (8%)	71 (92%)	0.7789
Extranodal localisation^2^	51 (30%)	8 (16%)	43 (84%)	0.0048
Constitutional symptoms	101 (60%)	10 (10%)	91 (90%)	0.0930
Bulky disease	35 (21%)	2 (6%)	33 (94%)	0.7046
Poor prognostic disease^3^	63 (38%)	7 (11%)	56 (89%)	0.1263
Haemoglobin level < 100 g/L	12 (7%)	1 (8%)	11 (92%)	0.8262
Pre-chemotherapy platelet count >350×10^9^/L	58 (35%)	6 (10%)	52 (90%)	0.2488
Pre-chemotherapy leukocyte count >11×10^9^/L	47 (28%)	5 (11%)	42 (89%)	0.2796
High KRS^4^	35 (21%)	2 (17%)	33 (21%)	0.7046
High ThroLy score^5^	7 (4%)	3 (43%)	4 (57%)	0.0002
Intermediate ThroLy score^5^	29 (17%)	4 (14%)	25 (86%)	
Low ThroLy score^5^	131 (79%)	5 (4%)	126 (96%)	

Abbreviations: KRS, Khorana Risk Score; MPV, mean platelet volume; ThroLy score, Thrombosis Lymphoma score; VTE, venous thromboembolism.

^1^ The percentages are related to the numbers given in the first column of the same line.

^2^ Extranodal involvment/advanced disease according to Lugano stage IV.

^3^ International Prognostic Score, high risk (3–7 points).

^4^ According to the Khorana Risk Score for VTE-risk assessment; High-risk group: 3–4 points.

^5^ According to the ThroLy sc ore; low (Score 0, 1), low (Score 2, 3) and high (Score > 3).

p < 0.05, statistically significant.

For the whole study group, the values of MPV were significantly lower in the HL patients (median 7.2, 25^th^-75^th^ percentile 6.8-8.3, range 5.79 – 9.4 fl) in comparison to the controls (median 7.95, 25^th^-75^th^ percentile 7.22-8.8, range 5.01 - 11.5 fl, p<0.0001), while the platelet count was significantly higher in the HL patients (median 313×10^9^/L, 25^th^-75^th^ percentile 264-392×10^9^/L, range 56-788×10^9^/L) in comparison to the controls (median 241×10^9^/L, 25^th^-75^th^ percentile 208-288×10^9^/L, range 129-501×10^9^/L, p<0.0001).

According to the Khorana Risk Score, 35 (21%) patients were considered as high risk of VTE development and 132 (79%) patients as intermediate risk, whereas according to the ThroLy score, 7 (4.2%) patients had a high risk, 29 (17.4%) patients had an intermediate risk and 131 (78.4%) patients had a low-risk of VTE development.

### Venous thromboembolism

In the whole study group, twelve (7.18%) patients developed VTE during first-line treatment in the median 1 month (25^th^-75^th^ percentile 1.0-3.3, range 1.0-6.0 months), including 6 (50%) cases of deep vein thrombosis of extremities, 1 (8%) symptomatic pulmonary embolism and 5 (42%) cases of internal jugular vein thrombosis. VTE occurred in 2 patients of the high-risk group (17%) and in 10 patients (83%) of the intermediate group according to the KRS classifications. More VTE events were found in patients with advanced stage IV (67% versus 28%, p=0.0048), and with bulky disease (mediastinal involvement) than without (50% versus 13%, p=0.0064). VTE occurred in 43% (n=3) of the high-risk patients and in 17% (n=2) of the intermediate-risk and in 4% (n=5) of the low-risk of the ThroLy score.

**Figure 1:**
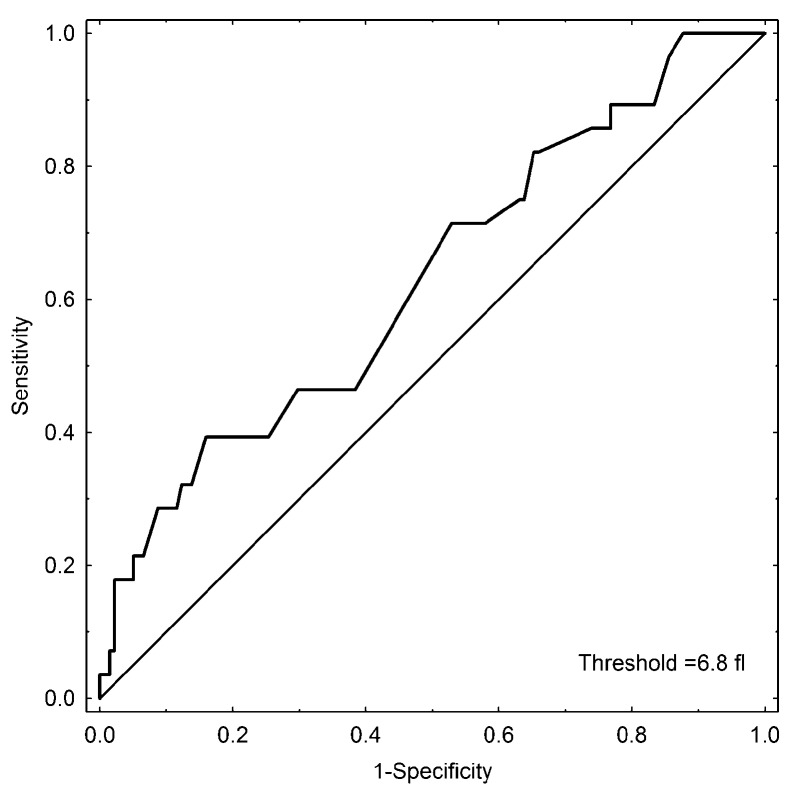
Receiver operating characteristic (ROC) curve analysis of MPV for the prediction of VTE development in HL patients

No difference in the pre-chemotherapy platelet counts between the patients who developed VTE during follow-up (median 361 x10^9^/l, 25^th^-75^th^ percentile 229-473 x10^9^/l, range 55-788) and the patients without VTE (median 312 x10^9^/l, 25^th^-75^th^ percentile 264-390 x10^9^/l, range 102-723 x10^9^/l, p=0.3871) was found. The pre-chemotherapy values of MPV were significantly lower in the patients who developed VTE during follow-up (median 6.9, 25^th^-75^th^ percentile 6.28-7.35, range 5.93-7.70 fl) in comparison to the patients without VTE (median 7.2, 25^th^-75^th^ percentile 6.90-7.80, range 5.79-9.40 fl, p=0.034). The ROC analysis indicated a cut-off value of 6.8 fl (95% CI 0.547-0.765) for MPV with 42% sensitivity and 79% specificity (ROC AUC=0.656, SE=0.056) for the development of VTE, Figure 1. The patients were therefore categorized using a cut-off set at the 25^th^ percentile of the overall population (6.8 fl). Four of the 21 (19%) patients with baseline MPV 6.8 fl or below developed VTE compared to 8 of the 148 (5.5%) patients with MPV values above 6.8 fl (p=0.0244).

**Figure 2:**
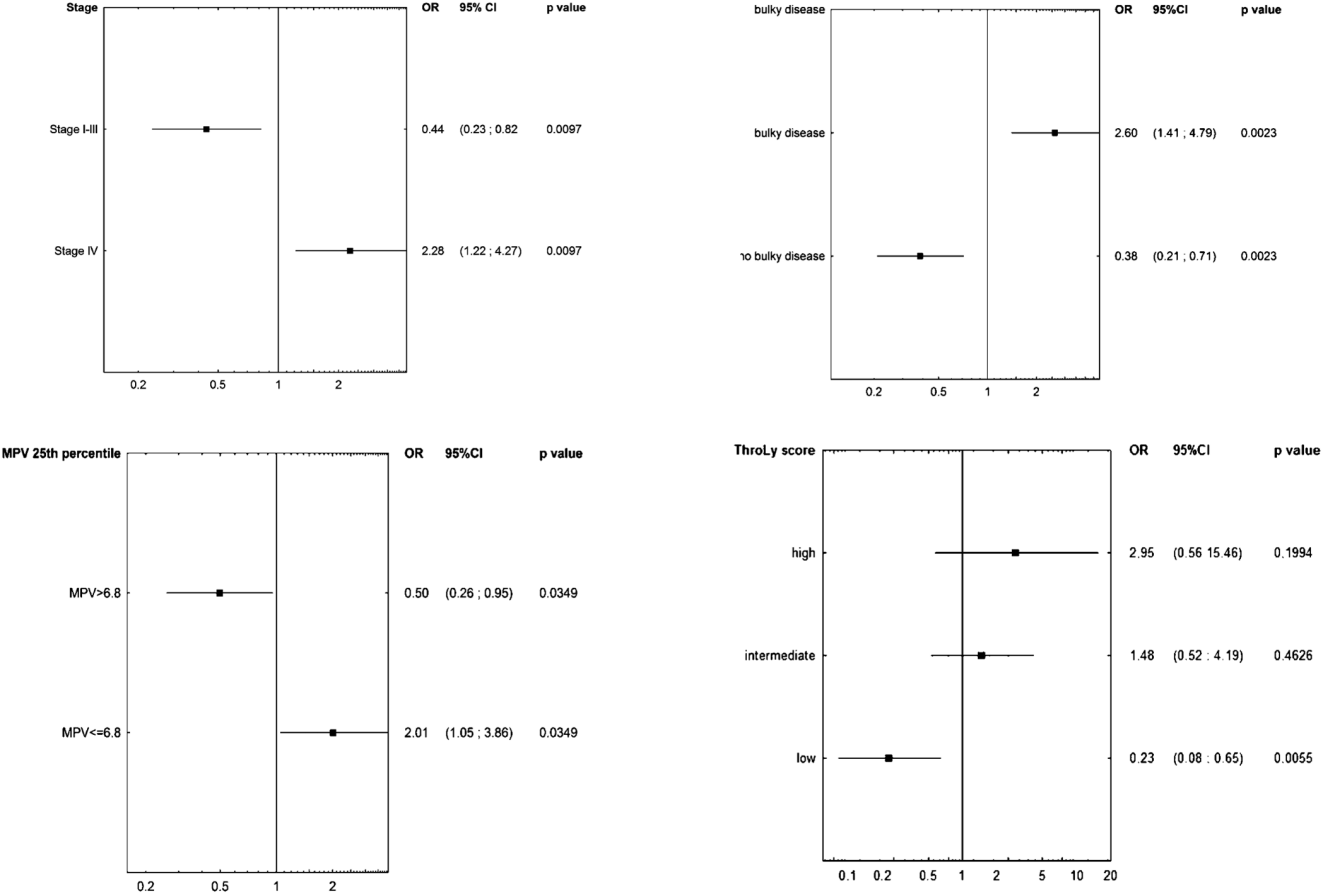
Univariate analyses determining factors affecting VTE development including **(A)** stage, **(B)** bulky disease, **(C)** MPV≤25^th^ percentile and **(D)** the ThroLy score. Abbreviations: CI, confidence interval; MPV, mean platelet volume; OR, odds ratio; ThroLy score, Thrombosis Lymphoma score.

In univariate analysis, MPV≤25^th^ percentile (OR 2.01; 95% CI 1.05-3.86, p=0.0349), advanced stage IV (OR 2.28; 95% CI 1.22-4.27, p=0.0097) and bulky disease (OR 2.01; 95% CI 1.05-3.86, p=0.0349) were associated with the occurrence of VTE, Figure 2. Other patient-related factors; age, gender, pre-chemotherapy platelet count over 350 × 10^9^/L, leukocyte count over 11 × 10^9^/L, haemoglobin below 10g/dl and disease-related factors; high values of International Prognostic Score, presence of constitutional symptoms or high risk in the Khorana VTE risk assessment model or high risk in the ThroLy score, failed to be prognostic for VTE. In multivariate analysis, MPV≤25^th^ percentile (OR 2.21; 95% CI 1.07-4.57, p=0.033) and advanced stage (stage IV versus stage I-III, OR 2.08; 95% CI 1.06-4.07, p=0.033) and bulky disease (OR 2.23; 95% CI 1.16-4.31, p=0.016) remained significant factors for developing VTE.

At the cut-off point for the high-risk category (score ≥3), we calculated the sensitivity (probability of high risk in those patients experiencing VTE), specificity (probability of high risk in those not experiencing VTE), positive predictive value (PPV, probability of high risk in those patients identified to be at high risk), and negative predictive value (NPV, probability of no VTE in those patients identified to be at low risk) for VTE development. For the KRS, the sensitivity was 100%, the specificity 0%, the PPV 100%, and the NPV 0% (ROC AUC/C statistic 0.523), whereas for the ThroLy score, the sensitivity was 33%, the specificity 80%, the PPV 67%, and the NPV 20% (C statistic 0.557). We expanded the Throly score by adding the MPV≤25^th^ percentile as 1 point, which gave an improvement in the C statistic (ROC AUC), which then reached 0.645. The sensitivity of the expanded model was 43%, the specificity 86%, the PPV 14%, and the NPV 57%.

### VTE-free survival

**Figure 3:**
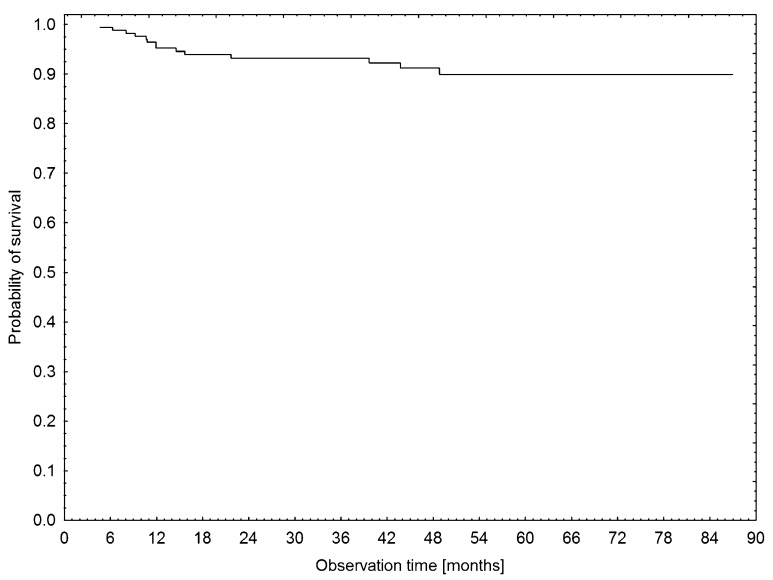
Kaplan–Meier estimates for survival probability of the patients with HL in the studied cohort

**Figure 4:**
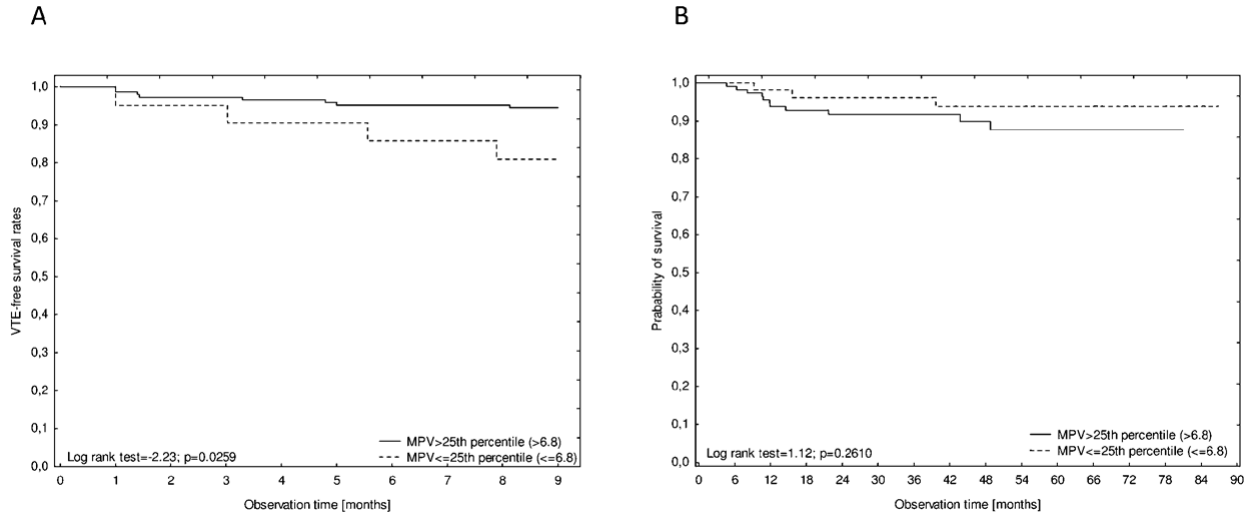
Kaplan–Meier analysis of VTE-free survival rates **(A)** and overall survival rates **(B)** according to pre-chemotherapy MPV≤25^th^ percentile or MPV>25^th^ percentile (6.8 fl).

**Figure 5:**
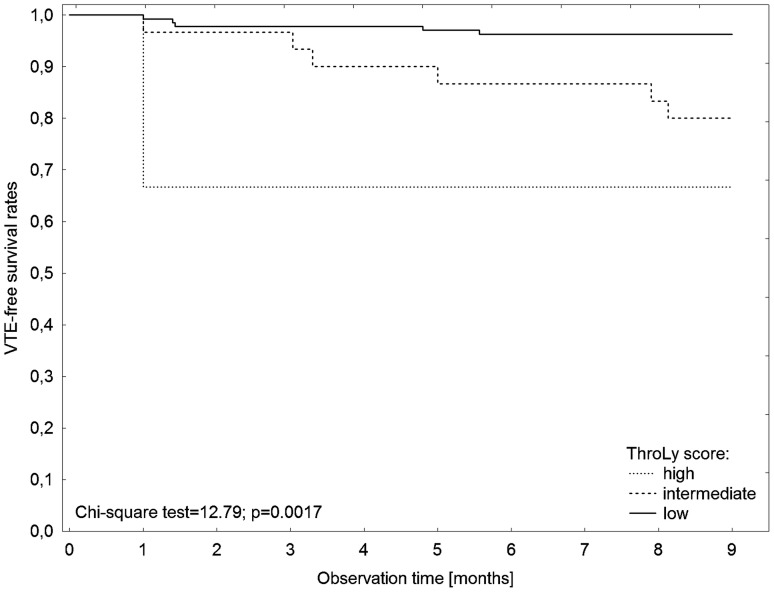
Kaplan–Meier analysis of VTE-free survival rates according to the ThroLy score

Figure 3 shows a cumulative survival plot for all analysed patients with HL. In a Kaplan–Meier analysis of the probability of VTE-free survival rates, patients with a low MPV (MPV≤25^th^ percentile) had significantly lower VTE-free survival rates compared to patients without low MPV (log rank test=2.23, p=0.0259), Figure 4A. Patients with bulky disease had, according to the Kaplan–Meier method, significantly lower VTE-free survival rates than those without bulky disease (log rank test=3.59, p=0.0003). In a Kaplan–Meier analysis of VTE-free survival rates, no difference was found between the patients in the high KRS and in the intermediate KRS group (log rank test=0.39, p=0.7004). In the Kaplan–Meier analysis, patients in the low-risk group of the ThroLy score had statistically significantly longer VTE-free survival than patients with a high- or an intermediate-risk score (Chi^2^ test=12.79, p=0.0017), Figure 5.

### Survival

During a median follow-up of 44 months, 14 patients (8%) died. None of the deaths were related to VTE. The Kaplan–Meier analysis did not show any difference in survival rates between patients with or without VTE (log rank test=1.026, p=0.3050). Moreover, no impact of the presence of bulky disease on overall survival rates was found (log rank test=10.1, p=0.9452).

In a Kaplan–Meier analysis of the probability of survival, no differences between the patients with pre-chemotherapy MPV≤25^th^ percentile and patients with MPV>25^th^ percentile (log rank test=1.124, p=0.2610; Figure 4B) and between the patients in the high KRS and in the intermediate KRS group (log rank test=0.1340, p=0.1803) were found.

In the Cox regression model, only high IPS (≥ 3 points) was significantly correlated with mortality (HR 6.10 (95%CI 1.20-30.98), p=0.0290), Table 2. Only a trend between the high ThroLy score and mortality was revealed. Other parameters including age, male gender, advanced disease, bulky disease (mediastinal involvement), presence of VTE, high KRS and MPV≤25^th^ percentile failed to have any impact on mortality.

**Table 2:** Factors affecting mortality according to the Cox proportional hazards model

Factor	Hazard Ratio (95% CI)	p value
Age	0.97 (95%CI 0.93-1.02)	0.2357
Male gender	2.04 (95%CI 0.59-6.99)	0.2572
Advanced disease^1^	0.84 (95%CI 0.18-3.86)	0.8245
Poor Prognostic Disease^2^	6.10 (95%CI 1.20-30.98)	0.0290
Bulky disease	0.17 (95%CI 0.01-2.20)	0.1730
High KRS^3^	1.04 (95%CI 0.29-3.76)	0.9594
High ThroLy score^4^	28.89 (95%CI 0.76-1098.2)	0.0561
Presence of VTE	2.24 (95%CI 0.60-8.42)	0.2291
MPV≤25^th^ percentile	2.63 (95%CI 0.65-10.52)	0.1737

Abbreviations: CI, confidence interval; HR, hazard ratio; KRS, Khorana Risk Score; MPV, mean platelet volume; ThroLy score, Thrombosis Lymphoma score; VTE, venous thromboembolism.

^1^ Extranodal involvment/advanced disease according to Lugano stage IV.

^2^ IPS, International Prognostic Score ≥ 3.

^3^ According to the Khorana Risk Score for VTE-risk assessment; High-risk group: 3-4 points.

^4^ According to the ThroLy score; low (Score 0 – 1), low (Score 2 – 3) and high (Score > 3).

p < 0.05 – statistically significant.

## DISCUSSION

To our knowledge, this is the first analysis to determine the association of MPV with symptomatic VTE occurrence in patients treated for newly diagnosed Hodgkin lymphoma (HL) and their mortality.

Venous thromboembolism is one of the major causes of complications and the second cause of mortality in cancer patients receiving outpatient chemotherapy, including Hodgkin lymphoma [5, 18, 19]. One of the key factors for VTE development is the platelets contribution to the clot formation [7, 8, 20]. The mean platelet volume (MPV), among other markers related to platelet functions and platelet number, is one of the most commonly available haematological parameters. There is evidence that MPV is a surrogate of platelet turnover, because larger platelets are younger and more reactive compared to their counterparts and the association between increased MPV with VTE and cardiovascular risks has been well established [21–24]. Contrary to these findings, in recently published studies, a lower MPV was associated with an increased risk of VTE and increased mortality in patients with heterogeneous cancer [9, 10]. Several studies indicate that the different types of malignancies and location had different thrombotic burdens [1, 6, 25], thus we sought to determine the association of MPV with symptomatic VTE occurrence in patients treated for newly diagnosed Hodgkin lymphoma (HL) and their mortality. In the present study, we demonstrated that patients with HL had lower pre-chemotherapy MPV levels compared to the healthy control group. Receiver Operating Characteristic curve was used to determine the optimal MPV cut-offs, which were similar to a previous study by Riedl et al., set at the 25^th^ percentile of the overall population [9]. Of the HL patients, in both the univariate and multivariate models, the patients with baseline MPV levels below the 25^th^ percentile had an above 2.0-fold increased risk of VTE development. Similarly, we previously reported the predictive values of low baseline MPV for a significant risk of VTE in patients treated for diffuse large B-cell lymphoma (DLBCL) [26]. These results are consistent with literature indicating the impact of low MPV on the risk of development of VTE in other types of malignancies [9, 10]. Further studies conducted by Riedl J et al. on the activation status of platelets in cancer patients both by flow cytometry and platelet activation markers, confirmed the association of decreased platelet reactivity with a high risk of VTE and poor prognosis, presumably as a consequence of continuous activation [27, 28].

In literature there is only one study designed to develop and validate a predictive model for thromboembolic events in lymphoma patients which is based on heterogeneous types of lymphoma patients, including a small number of HL patients (n=266, 14.6%). This is known as the ThroLy score [13]. In that study the MPV levels were not evaluated. In the present study, advanced stage IV of the disease and the presence of bulky disease were associated with approximately 2.0-fold increased odds for VTE occurrence. These findings are consistent with ThroLy score (extranodal localisation and mediastinal involvement) [13] and in accordance with the results of Lekovic et al. who found that patients with primary mediastinal large B-cell lymphoma had a higher incidence of VTE in the presence of a larger diameter of mediastinal tumor mass [29]. The data on the impact of a disease stage on VTE development in lymphoma patients are contradictory, in a meta-analysis by Caruso et al. only a tendency towards an increased rate of thrombotic events with more advanced stages of the disease was found [30], however, in other studies a higher stage of lymphoma has been associated with increased VTE risk [31–35]. Central venous catheters are known to increase the transient odds of VTE in lymphoma patients [36], these were not implemented during their first-line therapy of our cohort and therefore their impact could not be confirmed. None of the other studied factors, including gender, age, disease-related factors (poor prognostic disease and presence of constitutional symptoms) had a significant effect on VTE development. Although the Khorana Risk Score was developed for the stratification of cancer outpatients with different types of malignancies, and so far it has been best validated to stratify the risks of VTE [1, 20, 37]. Neither a high KRS nor a high ThroLy score could identify patients at a high risk group of VTE with a high degree of accuracy. Only 2 out of 35 of our patients in the high-risk of the KRS and 3 out of 7 patients in the high-risk group of the ThroLy score developed VTE. Consistent with literature, our data show that VTE events occurred more often in an intermediate than a high-risk KRS [38, 39]. Our results are in line with the results of several studies on other neoplasms in which KRS did not adequately stratify and predict VTE events in patients at high risk of VTE [40–42]. Although, Santi et al. reported that the KRS is predictive of VTE events among non-Hodgkin lymphomas including DLBCL [43], other studies failed to show any association of the KRS with VTE [26, 34]. The ThroLy score has not yet been validated by independent research. We found that incorporation of another marker, MPV≤25^th^ percentile, improved the discriminatory performance of the ThroLy score. Additional research could find yet more biomarkers that could further increase the accuracy of VTE-predictive models in lymphoma patients.

Contrary to the findings of previous studies reporting that cancer-associated thrombosis is a leading cause of death among patients with cancer and also in the lymphoma subgroup [2, 19, 32, 44], in our study cohort, no difference in the overall survival rate between patients with or without VTE treated for HL was found. Our results are in agreement with Lim S.H. et al. [34]. Moreover, in the present study, no impact of a low MPV nor a high KRS on prognosis was found. Our data are in line with a recent meta-analysis, in which no correlation between MPV and disease-free survival rate in patients with malignant tumours was found [45], however this is contrary to previous research [9, 26, 40, 46–49]. Reasons for such a divergence may include the relatively young age of our studied cohort, good performance status, limited observation period (median follow-up 44 months), no obesities and a low number of comorbidities. In the Cox model, only IPS 3 or above had a nearly 6-fold increased risk of mortality in HL patients [12]. None of the other studied variables were prognostic for inferior survival rates in our HL patients.

Although patients with lymphoma are considered to have a high risk of VTE development, with the incidence rate reaching 7.2% in our study, routine thromboprophylaxis to prevent venous thromboembolism is not recommended by current international guidance [50–52]. It has recently been shown that currently available prediction scores including the Khorana, Vienna CATS, PROTECHT, and CONKO performed poorly in predicting venous thromboembolism in cancer patients and the use of prophylaxis in outpatients undergoing chemotherapy remains a controversial issue [42, 53]. Therefore, our findings indicate the need for the identification of new biomarkers predictive of VTE, such as a pre-chemotherapy MPV≤25^th^ percentile, and for the improvement of lymphoma-specific VTE-assessment models.

The main limitation of our study is the retrospective collection of data. However, very limited original data have been published with regard to the MPV levels in lymphoma patients with or without VTE. Moreover, we evaluated only a Caucasian population. However, all patients were managed with the same procedure according to diagnosis and treatment in one hospital. There was no routine screening for VTE and only symptomatic events were evaluated.

In conclusion, in our study on patients with HL undergoing first-line therapy, advanced stage IV of the disease and the presence of bulky disease were associated with the development of VTE. Moreover, patients who had the baseline MPV≤25^th^ percentile, which is platelet-specific variable, also had a two-fold increased risk of VTE development. Our findings show that the incorporation of the MPV≤25^th^ percentile increases the discriminatory performance of the ThroLy score and there is a need for improvement of lymphoma-specific VTE-assessment models. Further larger prospective studies are needed to confirm or refute our findings before implication in practice.

## MATERIALS AND METHODS

### Patients

We retrospectively studied consecutive adult patients newly diagnosed with HL and receiving first line chemotherapy between 2009 and 2016, for VTE risk factors and the occurrence of VTE. All studied patients were in good general condition (ECOG/WHO performance status 0-2) and were qualified for ABVD regimen in the outpatient clinic of the Department of Haematology and Bone Marrow Transplantation at the Poznan University of Medical Sciences between June 2009 and June 2016. Administration of chemotherapy was by peripheral veins and no patients had central venous catheters implemented during their first-line therapy. None of the patients were given erythropoiesis-stimulating agents. The observation time was defined by the study end date (December 2016), disease progression, and occurrence of VTE or death.

Patients who received anticoagulants at the start of chemotherapy due to atrial fibrillation or previous VTE events were excluded from the study (n=7). No routine screening for VTE was conducted. Diagnosis of symptomatic VTE consisted of ultrasounds with Doppler and colour imaging for deep-vein thrombosis (DVT), or computed tomography angiography (CTA) for pulmonary embolism.

Demographic data and clinical details (stage of disease according to the Lugano classification, presence of constitutional symptoms, bulky disease (mediastinal involvement) defined as the longest measurement of tumour mass of 10 cm or greater, International Prognostic Score (IPS) and KRS) were all analysed [11, 12]. All demographic and clinical data for all patients were complete from the commencement of treatment and during follow-up. According to the KRS, patients were categorised into intermediate (1-2 points) and high risk (≥3 points) groups for the development of VTE, based on the site of cancer (lymphoma as high-risk), pre-chemotherapy platelet count over 350 × 10^9^/L, leukocyte count over 11 × 10^9^/L, haemoglobin below 10g/dl and/or use of erythropoiesis-stimulating agents, and a body mass index above 35kg/m^2^ (BMI, 1 point each) [1].

The lymphoma VTE-risk scores and categorisations to high risk (3 or more points), intermediate risk (1-2 point) and low risk (0-1 point) were performed according to the prognostic Thrombosis Lymphoma model developed by Antic et. al., known as the ThroLy score, which includes; previous VTE/acute myocardial infarction/stroke (2 points), reduced mobility (ECOG 2-4, 1 point), obesity (BMI > 30kg/m^2^, 2 points), extranodal localisation (1 point), mediastinal involvement (2 points), neutrophils below 1 × 10^9^/L (1 point) and haemoglobin level below 100g/L (1 point) [13].

For the MPV estimation and the KRS, a full blood count was performed by standard methods within 60 minutes of collection. The associations between the pre-chemotherapy MPV and VTE development and the outcomes were assessed. MPV values were evaluated in the control group. A control group consisting of 184 age-matched subjects was implemented. None of the control group had ever experienced symptoms of VTE or arterial thrombosis (acute myocardial infarction, peripheral arterial disease or stroke).

Furthermore, we expanded the ThroLy score for the prediction of VTE in lymphoma patients by adding one new biomarker, MPV, to find out whether the stratification of patients into high- and intermediate-risk group could be achieved more accurately.

The Bioethical Committee of Poznan University of Medical Sciences approved the study, in accordance with the Declaration of Helsinki.

The present report adheres to the Strengthening the Reporting of Observational Studies in Epidemiology (STROBE) Statement [14].

### Statistical methods

Assuming a VTE event rate of about 8% based on averages from literature [15–17], we calculated that at least 120 patients would be required to determine the role of MPV with a power of 90% using a two-side test at an alpha level of 0.05. The results are presented using methods of descriptive statistics, such as frequency (n), arithmetic mean (x–) and standard deviation (SD) for normally distributed variables. Otherwise, medians and the standard error (SE) with interquartile ranges (25 and 75 percentile) were used. The Shapiro-Wilk test was performed to assess normality. In order to compare differences between the groups, the chi-square test was used for categorical variables and the Mann–Whitney U test for continuous variables. Receiver Operating Characteristic (ROC) curve analysis was performed to determine the MPV cut-off values for the MPV level predictive of VTE development and for the evaluation of VTE-risk assessment models.

Univariate logistic regression was used to evaluate potential risk factors that might influence VTE. Multivariate analysis was performed with selected variables that were significant in univariate analysis (p<0.05). In each model, the odds ratio (OR) for each independent variable was determined with a confidence interval (CI) of 95%.

The probabilities of survival were estimated by the Kaplan–Meier method and univariate comparisons were performed using the log-rank test, otherwise the Chi-square test was used to analyse multiple variables. The Cox proportional hazards model was fitted to estimate the effect of the analysed factors on the outcome. In this model, the hazard ratio (HR) for each independent variable was determined with a confidence interval (CI) of 95%. A p-value below 0.05 was regarded as statistically significant. The statistical analyses were performed with STATISTICA 13 and STATISTICA Medical Package (StatSoft, Inc. Tulsa, Oklahoma, USA).
